# Systematic analysis of the relationship between fold-dependent flexibility and artificial intelligence protein structure prediction

**DOI:** 10.1371/journal.pone.0313308

**Published:** 2024-11-26

**Authors:** Neshatul Haque, Jessica B. Wagenknecht, Brian D. Ratnasinghe, Michael T. Zimmermann

**Affiliations:** 1 Computational Structural Genomics Unit, Linda T. and John A. Mellowes Center for Genomic Sciences and Precision Medicine, Medical College of Wisconsin, Milwaukee, WI, United States of America; 2 Clinical and Translational Sciences Institute, Medical College of Wisconsin, Milwaukee, WI, United States of America; 3 Department of Biochemistry, Medical College of Wisconsin, Milwaukee, WI, United States of America; Chung-Ang University, REPUBLIC OF KOREA

## Abstract

Artificial Intelligence (AI)-based deep learning methods for predicting protein structures are reshaping knowledge development and scientific discovery. Recent large-scale application of AI models for protein structure prediction has changed perceptions about complicated biological problems and empowered a new generation of structure-based hypothesis testing. It is well-recognized that proteins have a modular organization according to archetypal folds. However, it is yet to be determined if predicted structures are tuned to one conformation of flexible proteins or if they represent average conformations. Further, whether or not the answer is protein fold-dependent. Therefore, in this study, we analyzed 2878 proteins with at least ten distinct experimental structures available, from which we can estimate protein topological rigidity verses heterogeneity from experimental measurements. We found that AlphaFold v2 (AF2) predictions consistently return one specific form to high accuracy, with 99.68% of distinct folds (n = 623 out of 628) having an experimental structure within 2.5Å RMSD from a predicted structure. Yet, 27.70% and 10.82% of folds (174 and 68 out of 628 folds) have at least one experimental structure over 2.5Å and 5Å RMSD, respectively, from their AI-predicted structure. This information is important for how researchers apply and interpret the output of AF2 and similar tools. Additionally, it enabled us to score fold types according to how homogeneous versus heterogeneous their conformations are. Importantly, folds with high heterogeneity are enriched among proteins which regulate vital biological processes including immune cell differentiation, immune activation, and metabolism. This result demonstrates that a large amount of protein fold flexibility has already been experimentally measured, is vital for critical cellular processes, and is currently unaccounted for in structure prediction databases. Therefore, the structure-prediction revolution begets the protein dynamics revolution!

## Introduction

Recent advances in protein structure prediction have significantly expanded the fraction of the human proteome that can be modeled in high-resolution [1, 2]. Structural models enable researchers to better contextualize human genomic variations, predict lead compounds against previously un-characterizable active or allosteric sites, and more. This is a revolutionary step forward for the field. However, proteins require flexibility for function [3–5]. Thus, we sought to understand better the relationships among the multiple existing experimental structures for the same protein, how frequently they capture different functional states, and their comparison to predictions by AI algorithms. Specifically, we characterize how often AlphaFold2-generated models favor one specific state versus returning conformational intermediates or functional averages.

Large amounts of protein sequence and structural data are leveraged to drive AI-based structure prediction. The latest algorithms have found a balance between features datamined from evolutionary relationships apparent across protein multiple sequence alignments (MSAs) and non-linear 3D interactions from existing 3D experimental structures [1, 2, 6–8]. Evolutionary relationships include positions that co-evolve to preserve 3D contacts that underly structural stability or enzymatic function [9–11]. Additional advancements have emerged in the field, including large language models like MSA Transformer [12], EvoFormer [2], and Evolutionary Scale Modeling [13]. These developments have significantly enhanced the extraction of coevolutionary relationships among residues, leading to notable improvements in the prediction of 3D contact maps. Further, attempts have been made to reduce and randomize the amount of information input to AI systems to estimate protein flexibilities [14–16]. Then, experimental structural analyses determine crucial atomic details concerning amino acid packing, stability, biochemistry, and flexibility. Thus, there is critical information in both sequences and structures, and their clever combinations have powered the recent revolution in protein structure prediction.

AI-based protein structure prediction has revolutionized structural bioinformatics. The reliability of AI-predicted structures is now widely accepted [17, 18] and has rapidly changed research in many areas. For example, AI-predicted structures have improved the understanding of virus taxonomy [19], served as molecular replacement solutions where NMR-derived structures failed [20], and helped to complete models of challenging proteins such as thyroid-stimulating hormone receptor [21]. However, certain shortcomings of predicted structures have been discussed related to regions involved in ligand binding, higher flexibility, point mutations, post-translation modifications, and intrinsically disordered regions [18, 22, 23]. Bronstein *et al*. have shown that the role of extrinsic structural elements, like water, is sometimes indispensable for structural stability and is ignored in modeling [24]. Kaspers *et*.*al*., showed that the dynamics of multistate proteins are also ignored [25]. Jumper *et al*. noted that less common structural features like long β-sheets can be predicted well but may failed to produce the correct angle [26]. Thus, these revolutionary algorithms are not the final solution to the protein folding problem and do not capture protein dynamics. They should ideally be used with an appropriate understanding of their relative strengths.

**Fig 1:**
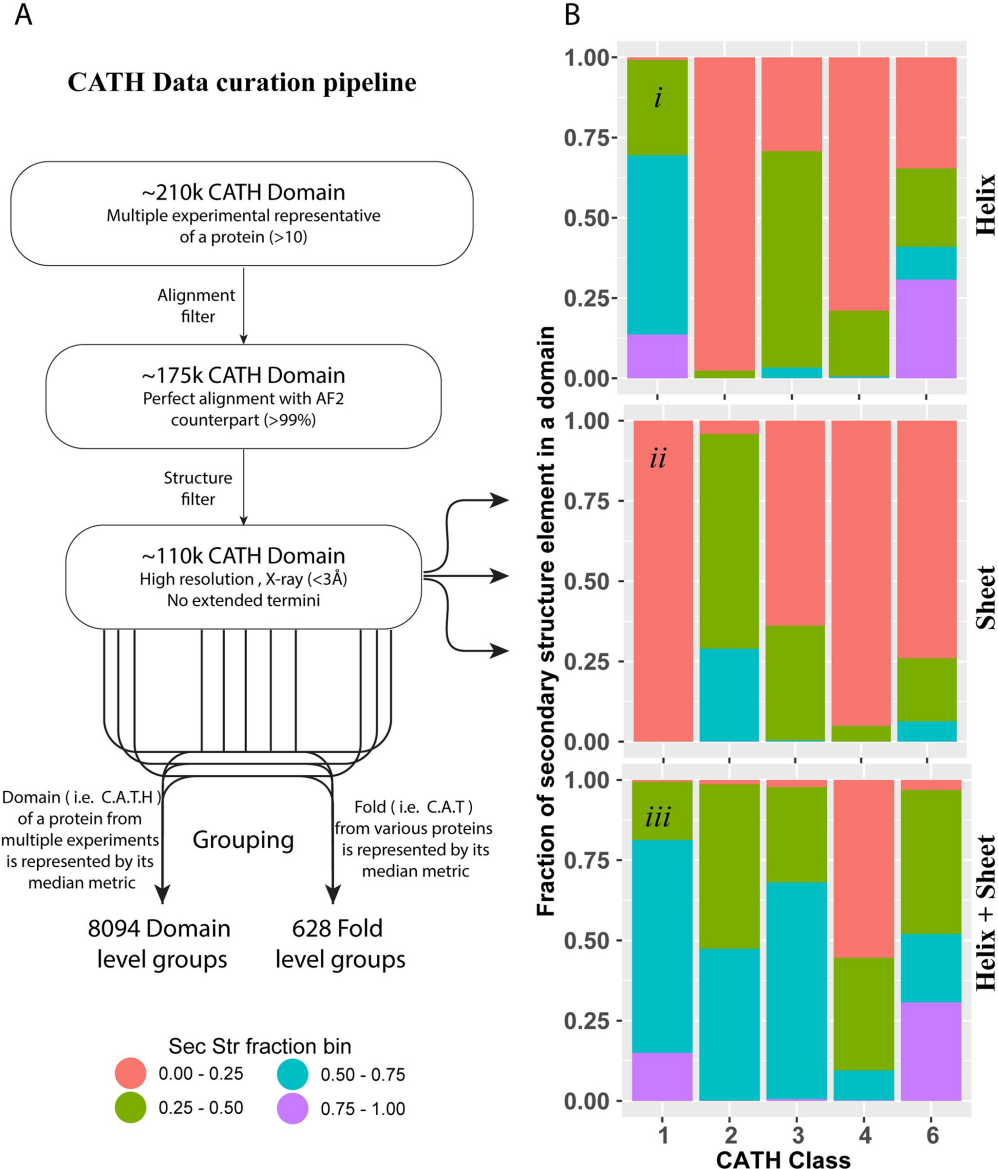
CATH data curation and the class characteristics. **A)** Depicts gross outline of data curation and grouping at domain and fold level. **B)** shows the fraction of *i)* Helix, *ii)* Sheet, and ***iii)*** Helix and Sheet combined across CATH classes. The secondary structures distribution was obtained from data (118,826 domain instances) before grouping. The grouping of data was performed at domain level (**C**lass, **A**rchitecture, **T**opology/fold, and **H**omologous superfamily), returning 8,094 domain groups. At the fold level (**C**lass, **A**rchitecture, **T**opology/fold) 628 groups were obtained.

Many proteins require flexibility for function, often manifest as multiple conformations across experimentally solved structures [27–29]. Consequently, each conformation may contribute to evolutionary coupling in the MSAs and 3D contact data used to train AI systems. Proteins are modular with their structures frequently constructed using archetypal folds. Knowledge of the inherent flexibility of each fold type is important for understanding the biologic function of proteins that contain each fold type. In this study, we seek to characterize the experimentally derived flexibilities of each fold type by measure the intra-domain conformational differences and how they compare to the predicted structures generated by the AI-based predictive tool Alphafold2. To achieve this goal and be precise in our evaluations, we focus on 176,257 protein domain instances with multiple existing high-resolution experimental conformations. Multiple experimental structures of the same protein, derived from different conditions, give a practical sampling (albeit incomplete) of the conformational diversity necessary for function. Recent works have demonstrated that AI-generated structures are discrepant from experiments in local details [30, 31]. Therefore, we took a next step from this data to characterize whether AI-generated models favor specific conformations or return functional averages that balance the 3D contacts and co-evolutionary constraints across conformations. We compared structures using four levels of resolution: individual experimentally determined protein domain instances, sequence-based clusters subdivided within CATH topologies (a.k.a. fold types; **S1 Fig** in S1 Text), all domains within the same CATH topology (**Fig 1A**), and the CATH class that each topology is part of. Our analyses reveal that current AI-based algorithms reproduce one specific state with high accuracy, on average, yet the pattern depends on the protein fold type. These results are essential for interpreting AI-based predicted structures’ strengths and limitations in further research.

## Methods

### Harmonizing and characterizing experimentally derived protein structures

We gathered sets of experimentally derived protein structures. The multiple experimental structures of the same protein in different conditions gives a sampling of the flexibility and conformational diversity necessary for protein function. Mappings between the PDB [32], CATH [33], and UniProt [34] were taken from the SIFTS [35] resource. We count unique experimental structures by distinct PDB IDs, individual proteins by UniProt Accession numbers, and unique folds as different CATH topologies.

We focused on understanding the structural similarity of the domains with their AI-predicted counterparts. We selected 5,388 proteins from the PDB that each have at least ten distinct experimental structures. Then, we merged the dataset with CATH annotations, leaving 3,870 distinct proteins with defined structured domains. Next, 213 proteins (5.5%) were excluded because no model was available from the AlphaFold2 (AF2) Protein Structure Database (ebi.ac.uk), leaving 3,657 proteins. Because each protein may have multiple distinct domains, this stage of the dataset contains 217,402 domain instances. A subset of domains’ sequences (defined by CATH) did not align well with their corresponding protein sequence obtained from EBI (e.g. due to mutations, typically to increase solubility). Therefore, we removed 37,721 domain instances with percentage sequence identity (PID) < 99, ignoring gaps in PID calculation. We also removed domain instances with ten or fewer amino acids. We further eliminated structures with extended N- or C-terminals and structures with lower resolution (>3Å). Our analyses were performed on a final dataset comprised of 2,878 proteins that contain 118,826 domain instances. This resource contains proteins from multiple organisms, with the human subset containing 1,288 (44.7% of the dataset) proteins. Sequence alignment was performed using BLOSUM62 substitution matrix, gap opening penalty of 10, gap extension penalty of 0.2, and the local-global hybrid algorithm of Needleman-Wunsch and Smith-Waterman algorithms as implemented in the pairwise Alignment function of the Biostrings R package [36]. A schematic illustration provides a succinct overview of our data filtration process (**Fig 1A**). Stringent data curation measures were implemented to minimize undesired features. To further mitigate the risk of overrepresentation of highly populated members, which could potentially distort data distribution and introduce bias in interpretation, two additional datasets were derived from our main dataset. The first additional dataset involved grouping similar domains from the same segment of a protein, represented by the median values of metrics such as RMSD, surface area ratio, and the ratio of buried and exposed residues. The second additional dataset was generated by grouping similar folds, defined by specific patterns and content of secondary structures as classified by CATH topologies, and is again represented by median metric values (see **S2 Fig** in S1 Text for additional details).

### Extracting domain instances from AI-predicted structures

Domains are typically the structural and functional units of proteins. An ideal canonical domain has a modular and specific shape, defined by the spatial orientation of secondary structure elements. The inter-domain regions of proteins are often more flexible than the domains. AF2 provides a full-length predicted model of each protein. The domain boundary definition provided with CATH is consistent with the PDB sequence but sometimes not with the UniProt reference sequence. We first extracted each sequence from the CATH domain to harmonize domain sequences. Then, we aligned each with the corresponding sequence of the AF2 full-length protein (by definition, also the UniProt canonical protein reference sequence). After alignment, we extracted all the residue segments from predicted structures, which aligned one-on-one with each protein’s reference sequence, producing the specific AF2-predicted domain conformation for each CATH domain. We use this dataset to calculate the RMSD, normalized RMSD100 [37], and FATCAT RMSD (S2-S4 Figs in S1 Text) between experimentally solved and AI-predicted domain conformations.

During our analysis, we observed high RMSD values for some domains due only to extended N-terminal or C-terminal regions despite virtually perfect structural alignment of the domain core. Therefore, we determined domain structures with extended N- and C-terminus and eliminated them from our dataset (see **S1 Text** for more details). We also observed limitations regarding the specific sequence-level differences in how domains are annotated to proteins, that complicated structure-based comparisons. Therefore, we made further domain subdivisions which go beyond the CATH ontology (see **S1 Fig in S1 Text**). Briefly, we defined topology sub-clusters that uniformly correspond to the same sequences: 1) when the same region of a protein was simultaneously annotated to multiple topologies, each is its own sub-cluster and 2) when a topology is annotated to a region of a protein, but different experiments resolved different windows of amino acids, each is its own sub-cluster.

### Structural metric values of the experimental domains

We calculated structural properties, including surface area to volume ratio (AVratio), buried residue to exposed residue ratio (BEratio), count of residues in contact with metal ions (nMet), count of residues in contact with ligands (nLig), and secondary structure content of the experimental and predicted models. We then used distributional comparisons to understand how these properties relate to structural deviations between experimentally solved and AF2-predicted structures (see **S1 Text** for more details).

## Results

### Domain classes have high variation in composition

#### Secondary structure diversity within domain classes

The domain class is the first organizational layer in CATH, which classifies proteins based on their secondary structures and how those secondary structures are spatially arranged. CATH defines six domain classes with a semi-definite proportion of secondary structure types. Analysis of our dataset suggests a more diverse balance of secondary structure content within each domain class. Class 1 is defined as mainly α-helix content, while 28.6% of the domains in this class have <50% α-helix content (**Fig 1B*i***). Similarly, Class 2 is defined as mainly containing β-sheet structures, yet we observe that 73.3% of the domains in this class have <50% β-sheet content (**Fig 1B*ii***). Class 3 is mixed α/β, and 33.6% of domains have <50% of classic secondary structures, while Classes 4 (unstructured) and 6 (non-globular) have 32.8% and 5.7% of the domains with <25% secondary structures (**Fig 1B*iii***). The proportion of domains in each class with low and varied secondary structures impacts how the individual domains are compared.

#### Differences in secondary structure content between AAF2-predicted and experimental structures

**Fig 2:**
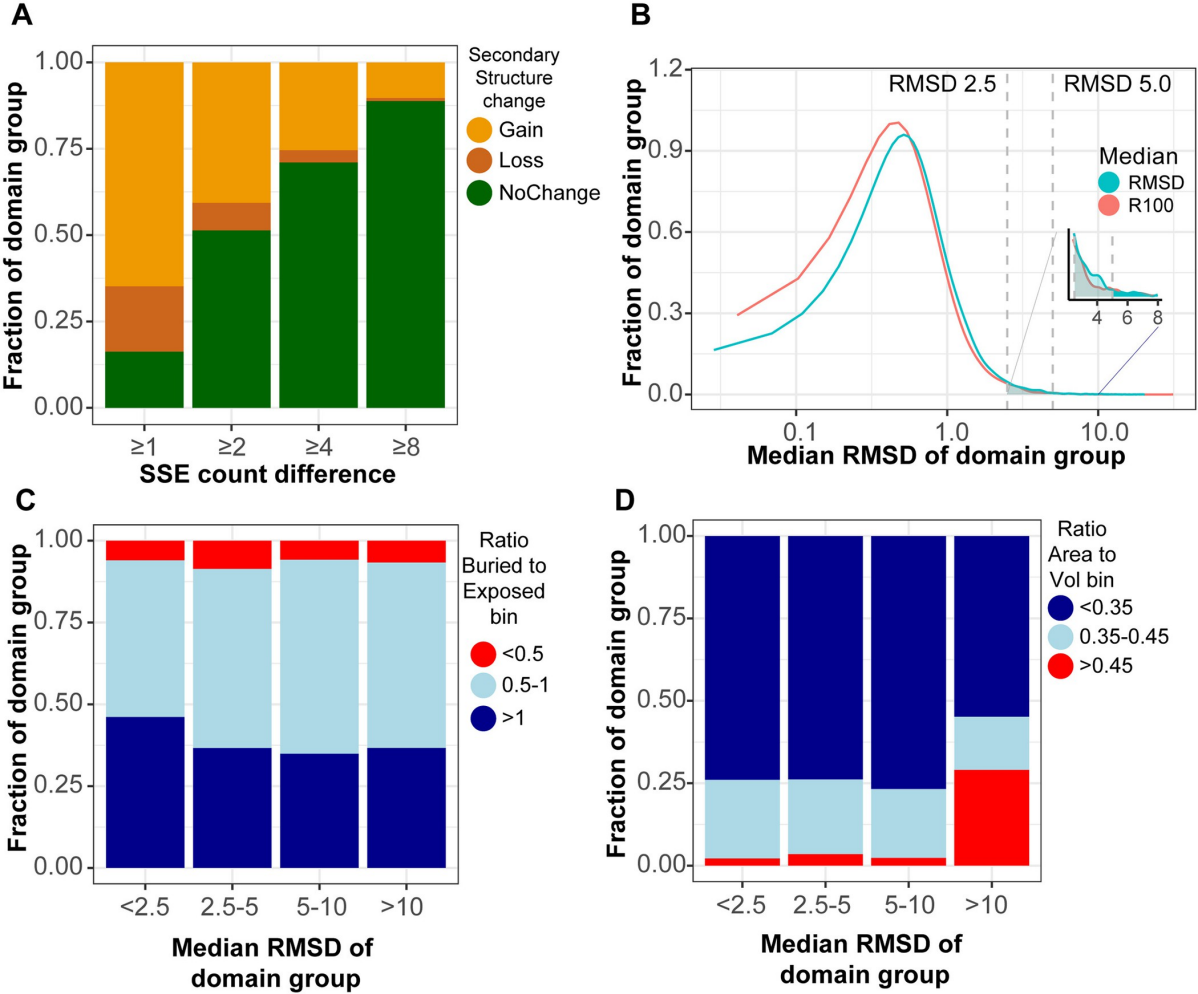
Domain group-wise distributions reveal geometric patterns. **A)** The distribution of SSE (secondary structure elements of helix and sheet) count difference with increasing difference cut off between experimental (CATH) and predicted (AF2) structures. **B)** Domain group distribution with respect to the median RMSD and median RMSD100 (shown as R100) of the group. **C)** The distribution of the median ratio of buried to exposed residues is consistent across the range of conformational differences. **D)** Interestingly, the median ratio of surface area to volume is consistent across the lower range of conformational differences, yet for the highest tier the conformations more frequently have a high ratio.

AF2 is a robust algorithm for protein structure prediction from merely the sequence; it produces an accurate structure of near experimental resolution. However, during the multistep process it has been known to overestimate the secondary structure content of the model [38]. Therefore, we computed the fraction of α-Helix and β-Sheet differences between experimental structures and their AF2-generated models in the current dataset. We observe more residues within secondary structure for predicted structures with high confidence (AF2 dataset filtered to structures with 90% of residues with pLDDT > 80). At least 64.80%, 40.70%, 25.50%, and 10.30% of domains have more than 1, 2, 4, and 8 additional residues in α-Helices or β-Sheets, respectively (**Fig 2A**). Yet, there are few instances of lower secondary structure content. Thus, the AF2-predicted structures may predict more secondary structure content compared to experiments, suggesting overestimation of local interaction prediction by the models.

### Global distribution of fold-specific conformational variation

#### Folds of most proteins are predicted to high accuracy

While the high accuracy of AlphaFold2 (AF2) predictions is well-established, the performance of AF2 in predicting dynamic domains has yet to be assessed. We quantify the extent of agreement between experimentally determined domain structures and their corresponding AF2-predicted structures using RMSD-based calculations. The predicted structures are within 2.5Å RMSD for 92.04% of domain instances. Nearly 97.67% of the domains have structural deviations below 5Å (**Fig 2B**). RMSD100 is normalized by protein length, (see **S1 Text**); it has similar global distribution to standard RMSD in our dataset. The domains with high RMSD are distributed among all domain topologies, folds, and even protein-specific domain group. The distribution shape is robust to accounting for and filtering out hinge-like motions, yet some of the largest conformational differences are hinge-like (**S3 Fig**). Thus, AF2-predicted structures are in good agreement with most experimental structures, yet with a considerable degree of intra-domain flexibility not represented.

#### The extent of conformational variation is fold-specific

Biologic systems use each fold to different extents, reflected in the current dataset. The dataset contains representatives from 628 distinct folds or domain topologies and 118826 individual domains, where 99.20% (n = 623out of 628) and 99.68% (n = 626 out of 628) of the folds have ≤2.5Å and ≤5.0Å RMSD for at least one member of the fold, respectively. The most populated folds are Rossman and immunoglobulin, with 14785 and 8052 instances, respectively (**S1 Table**). These two folds have as many experimental structures (19.21% of the dataset) as the 525 rarest folds together. Most folds with the highest median RMSD to predicted structures have few individual domains in the dataset. For example, of the 11 folds with median RMSD > 2.5Å, only two have at least 100 domain instances, and of the 37 folds with median RMSD > 1.5Å, only 6 have at least 100 domain instances (**S1 Table**). Yet, well-populated folds are among the most variable. For example, the HSP90 fold has 265 domain instances with median ± MAD RMSD of 6.49 ± 3.1Å, the Retinoid X Receptor domain has 1493 cases with 0.95±0.28Å, and the Zincin-like fold has 55 cases with 1.90±1.28Å. These examples underscore that certain folds, delineated by their topological architectures and classified within the same fold type, exhibit disparate responses to the AF2 algorithm. This observation suggests that the sequences within such folds possess the propensity to adopt divergent conformations.

#### Domain level grouping demarcates clear boundaries among domains of different nature and sizes

We identified a common challenge in RMSD calculations: unresolved residues in experiments and overlapping domain segments made ambiguity in precisely which amino acids are comparable. To solve this challenge, we first clustered intra-domain sequences at 95% identity using CD-HIT [39] and obtained 6087 distinct clusters of the 118826 input domains. Therefore, instances of the same domain topology in different clusters will have sequence differences (**Fig 1A & S1 Fig in S1 Text**). Second, we identified that sequences within a cluster had variable lengths and could belong to other (often overlapping in sequence) domain topologies (See **S3 Fig in S1 Text**). Thus, the members of sequence-based clusters were further clustered using a hierarchical method to resolve both features, producing 8094 sub-clusters. Each sub-cluster comprises domain instances of a specific topology and resolved sequence length with mutually ≥95% identity (**S1 Fig in S1 Text**).

The number of individual domain instances in each sub-cluster ranged from one to 628. The AF2 predicted model of each domain is ≤2.5Å RMSD for at least one experimental structure in 95.35% of the domain sub-clusters (n = 7718 out of 8094), and under 5Å RMSD in 98.78% (n = 7996 out of 8094) of domain sub-clusters. However, a large subset (89.10%, n = 7211 out of 8064) of the sub-clusters have all their members under 2.5 Å RMSD. Thus, 10.91% of the domain sub-clusters have a large conformational heterogeneity which produces variations between predicted and experimental structures for all their members. This strongly suggests a non-rigid and dynamic nature for these domain types.

### Conformational heterogeneity produces differences in geometric properties

Proteins belonging to the same fold can have different structural properties, including their enclosed molecular volume and surface area. Therefore, we analyzed each fold type’s relationships among structural variations and molecular geometric properties. The fraction of buried versus exposed amino acids (BE) ratio is high for folds with median RMSD within 2.5Å from AF2 structures and lower for higher median RMSD values (**Fig 2C**). This indicates that compact globular folds have more of their experimental structures in close agreement with predictions compared to more extended folds. A similar monotonic trend was found for the surface area-to-volume (AV) ratio, where a high AV ratio occurs with high median RMSD values and vice versa (**Fig 2D**). These observations are both robust to accounting for hinge-like motions (**S4 Fig in S1 Text**), which can explain only a small fraction of the inter-domain motions in our dataset. Interestingly, the presence of interacting metal ions and ligands did not follow a systematic pattern (S1 Text). This suggests a clear yet fold-dependent relationship between domain compactness and the apparent agreement with predictions.

### AI-predicted protein structures are highly specific to a single experimental state

We next characterized the fold-specific heterogeneity across all domain topologies and then highlight details of two supporting examples of specific domain topologies.

#### Each domain topology has a specific distribution of conformations that define a heterogeneous spectrum

**Fig 3:**
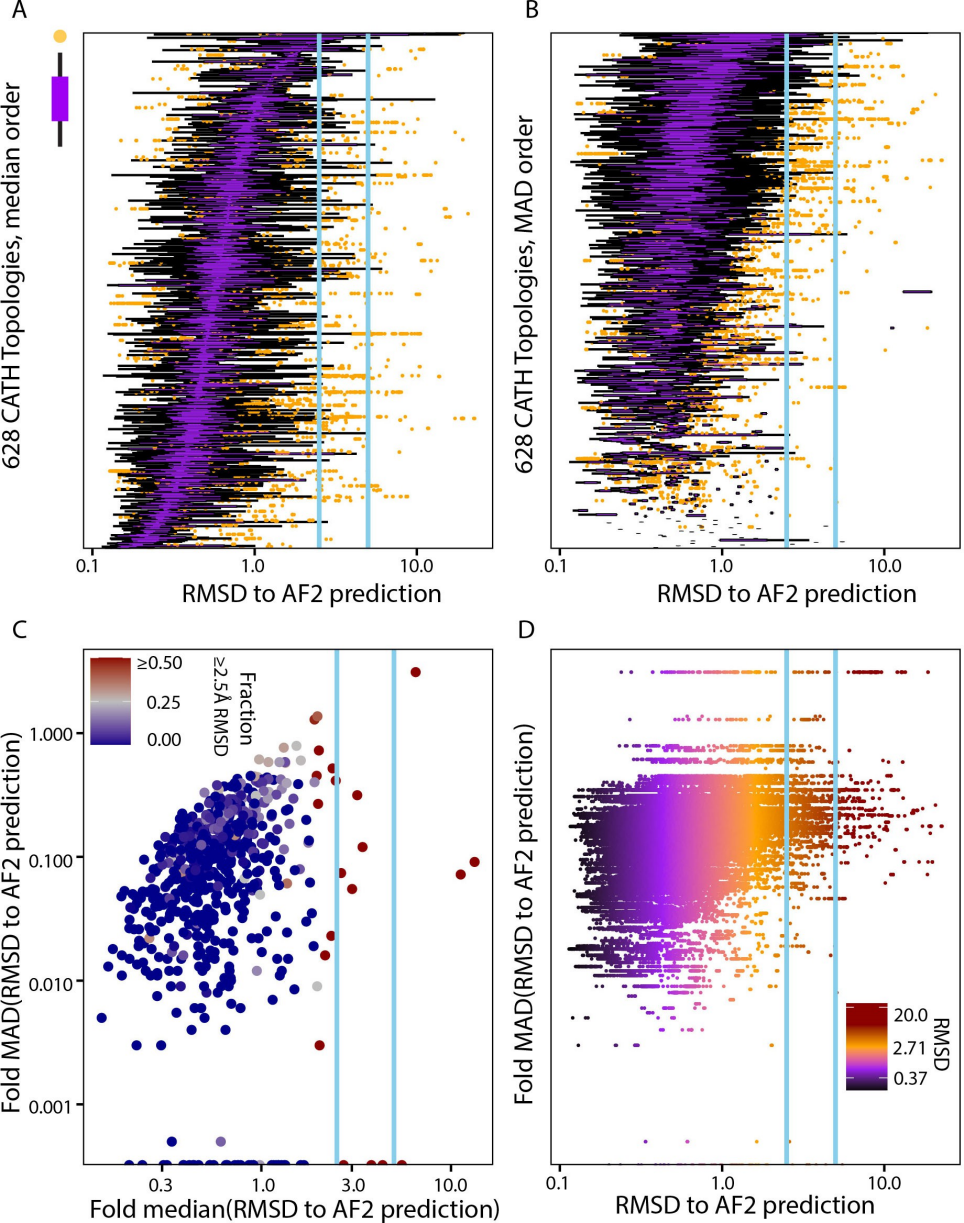
Protein folds exhibit a spectrum from conformationally homogeneous to highly heterogeneous. At the same time, AF2-generated structures represent specific structural conformations. We quantified the distribution of RMSDs to AF2-generated protein structure prediction on a per-fold basis. A and B) are ordered distribution of domain in fold, by median and MAD, a robust standard deviation, respectively. Each horizontal arrangement of dots is the box plot representation domains RMSD distribution where each dot represents domain RMSD. A is ordered by median and B is ordered by MAD values. The color of the horizontal dots, in the box plot, on the other hand shows inter quantile range (IQR) of the box plot (1*IQR purple, 1.5*IQR black), such representation makes it visually evident that even the most variable folds have a small number of instances that closely match experimental structures. **C)** Each fold has a robust average along a spectrum that loosely correlates with their level of variation. **D)** Each individual experimentally solved protein domain is shown as a point along the corresponding fold’s MAD to show conformational diversity in more detail.

Next, we define the spectrum of fold-dependent and experimentally derived conformational variation. Especially for heterogeneous folds, any individual predicted structure will always be a functional snapshot. We summarized the entire dataset by fold type as a census of the fold-dependent heterogeneity already captured by experimentally solved structures (**Fig 3A**), and which we anticipate reflects biologically relevant flexibility. We found that variation within a fold is mainly independent of the fold having a conformation that is predicted to have ultra-high accuracy (**Fig 3B**). However, a significant fraction (8.60%) of folds have more than 10% of their members ≥ 2.5Å RMSD from the predicted structure (**Fig 3C**). Most folds show a continuous spectrum of RMSD (**Fig 3D**), ranging from ultra-high consistency (e.g., < 1Å RMSD) to extremely divergent conformations (e.g.,> 10Å RMSD). Interestingly, the database is almost equally split between liganded (49.6%) and un-liganded protein domains; 0.98% of the domains are derived using NMR, the average crystallographic resolution is 2.3Å, and 15.6% of domains are comprised of more than one linear sequence segment; these features are independent of the fraction of domains that are ≥2.5Å RMSD from AI predictions (not shown). Thus, there is a broad need for interpreting the functional flexibilities of domain types, yet the existing experimental structures can provide a fold-dependent baseline.

#### RAS GTPases as exemplars for conformational heterogeneity focused to functional movements

We now focus on a specific example of a critical disease gene and RAS family GTPase, KRAS, that is comprised of a single fold type. KRAS oscillates between open and closed states at two switch regions. These switch regions depend on thermodynamic stability and ligand (GTP or GDP) binding. When the switches are folded against the protein, KRAS can bind to effector proteins and propagate cellular signaling cascades. Being a key signaling protein in cancer, we investigated how the many existing experimental KRAS structures compared to AI predictions, and how conformational heterogeneity within an individual fold type can drive biological phenomena.

**Fig 4:**
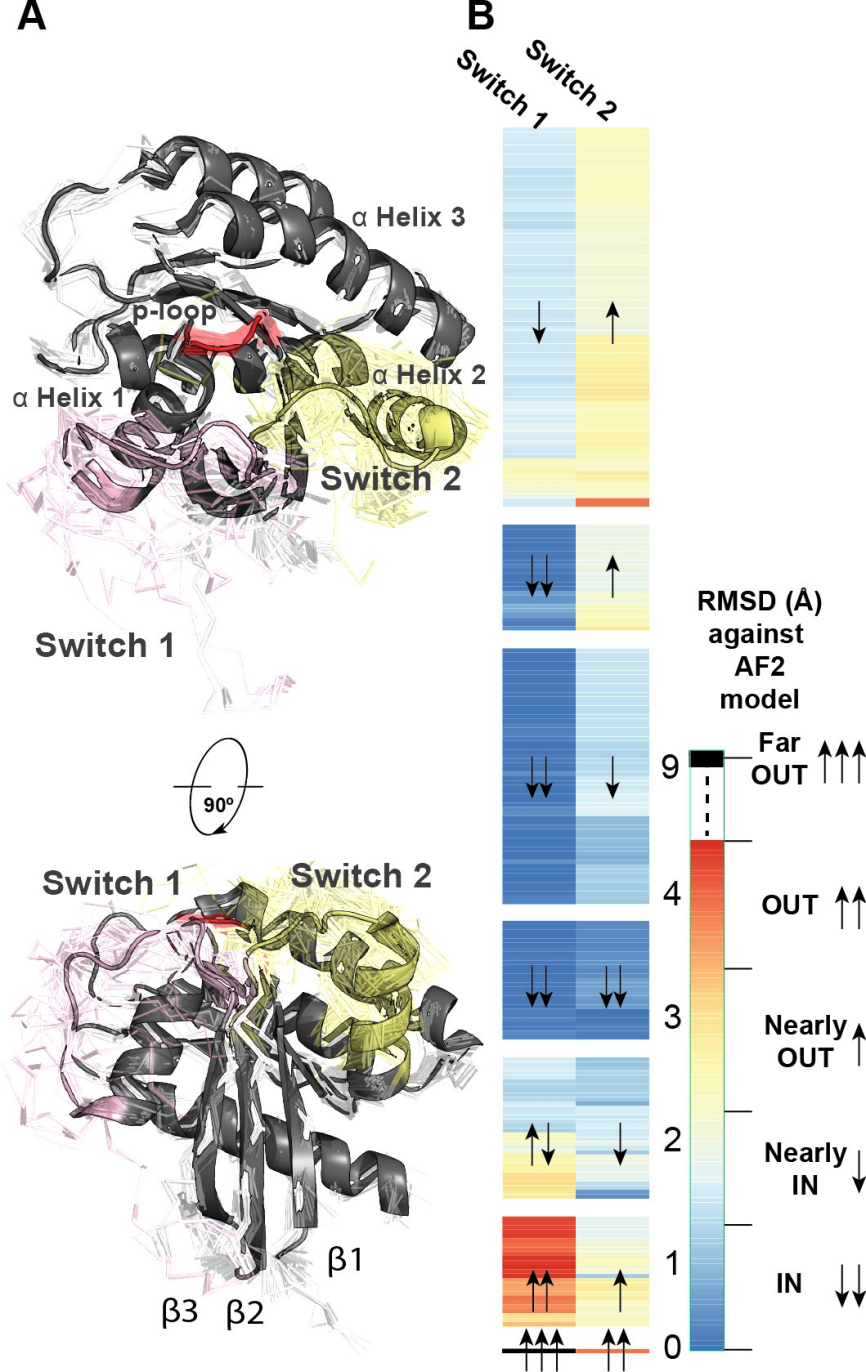
RAS experimental structures span a diverse range of switch conformations. **A**) Seven representative human KRAS structures, one from each cluster, are shown with important structural components highlighted. The predicted structure is shown in grey cartoon representation. Experimental confirmations are displayed in ribbon. The p-loop is colored red; Switch 1 is colored pink, and Switch 2 is colored yellow. **B**) Two-dimensional RMSD values of switch I and switch II, with respect to its corresponding AF2 aligned and subsequently extracted structures, clustering of experimental KRAS structures, represented by CATH 3D model. The clustering is described as a heatmap where the left and right columns correspond to switch I and II. Almost all the RMSD values are spread between 0.28 and 4.43Å. The heatmap was annotated with upward and downward-facing arrows to show the similarity of switch conformations of each cluster with respect to AF2. In the legend, we show a scale break to the single “far out” conformation (6BOF; 8.95Å RMSD for switch I and 3.83Å for switch II).

To better understand KRAS conformational heterogeneity, we studied its 253 existing experimental structures. Owing to its flexibility, both the switch regions (**Fig 4A**) are partially missing from 23 structures. We calculated RMSD values to the AF2-predicted model for switch I (residues 26–46) and switch II (residues 51–78) regions. The AF2 model has both switches in closed conformations where they form clear secondary structures. We observed diverse switch conformations sampled by experiments, while non-switch regions are more homogeneous (**Fig 4A**). We clustered the switch RMSD values for all 252 structures and identified six patterns (**Fig 4B**). Three of these patterns maintain switch-I in a close position, with three levels of switch-II movement. Two patterns have switch-I-in with switch-II-out and vice versa; the final pattern positions both switches out and lacks secondary structures. Nearly 14.62% of structures were under 1Å RMSD to the AI-predicted structure, and 75.5% were under 2.5Å of RSMD for both switches. Only 24.5% of the structures were above 2.5 Å of RSMD, and only one was above 5 Å of RSMD (**Fig 4B**). Thus, results suggest that the predicted structure of KRAS is in remarkable agreement with the experimental structures and represents the active conformation for cellular signaling. However, one-fourth of experimental conformations are divergent from the AI-predicted structure. These conformations are essential for the regulation of KRAS cellular signaling. Thus, well-predicted and well-characterized fold types like small GTPases are among the conformational heterogeneous folds, with their flexibility focused on critical regulatory motions.

#### Small molecule transporter domain examples predicted as functional intermediates

**Fig 5:**
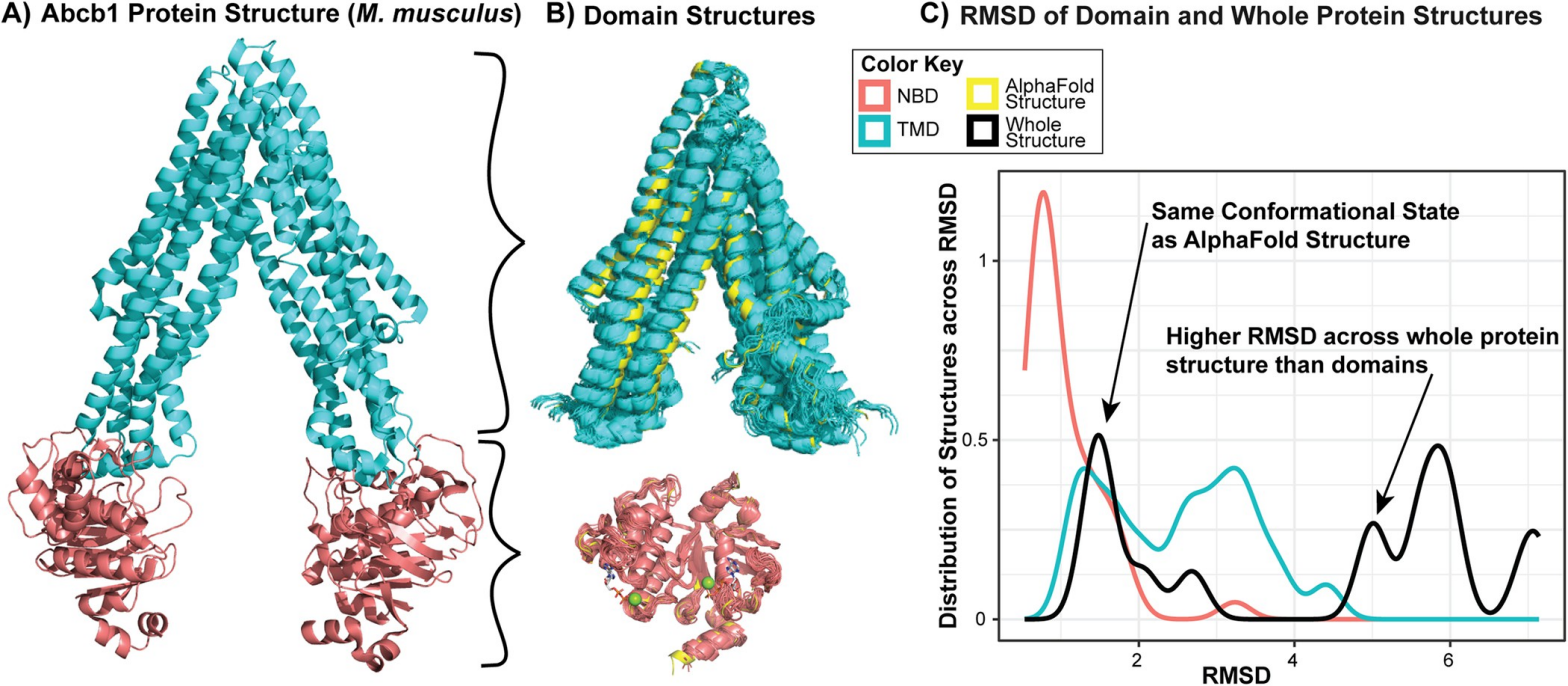
ABC family proteins’ ATPase domains have high conformational consistency while transmembrane domains have high heterogeneity to facilitate transport. **A)** One experimental structure of Abcb1a, the mouse P-glycoprotein (PDB ID: 5KPJ), is shown with the nucleotide binding domains (NBDs; CATH 3.40.50.300) colored pink and transmembrane domains (TMDs; CATH 1.20.1560.10) colored cyan. **B)** All experimental domain structures are shown aligned with their respective AF2 structures, shown in yellow. There’s a range of conformations shown in the experimental TMD structures, as the protein physiologically opens and closes to facilitate ligand transport. **C)** Whole protein RMSD is higher than RMSD of domains. NBD RMSD is extremely low, but some experimental TMD structures have higher RMSD from the AF2 structure due to their wider conformation. However, whole protein RMSD is much higher than that of either domain, demonstrating the accuracy gained by using domain structures rather than whole protein structures.

Having characterized KRAS, a small single-domain protein, we next sought a specific example for a multi-domain protein. We chose ATP Binding Cassette (ABC) protein B1 (ABCB1, also known as P-glycoprotein) as an example because of its impact on the pharmacokinetics of many drugs, including many cancer treatments, and because many experimental structures exist for its mouse ortholog, Abcb1a (**Fig 5A**). Additionally, despite how ubiquitous ABC proteins are and their significant pharmacokinetic impacts, many family members lack experimental structures. Therefore, predicted structures are of high value for supporting research in ABC transporters.

ABC family proteins transport substrates across membranes, facilitated by ATP binding and hydrolysis. Each of these functions is performed by a different fold type. The transmembrane domain (TMD) spans the plasma membrane and facilitates substrate transport. One nucleotide-binding domain (NBD) is attached to each end of the intracellular TMD. Many ABC proteins encode all three domains in one monomer, but some family members form from homodimers [40]. In either case, the resting state (State 1) is typically open to the intracellular side. When a ligand binds in the pocket within the TMD, it brings both NBD pairs together through TMD hinge motions (State 2). ATP binding between both NBDs further brings the TMD sides together to form the outward-facing conformation (State 3) and to complete ligand transport. ATP hydrolysis releases the outward-facing conformation and resets the process [41].

Upon visually inspecting the 21 TMD experimental structures and 42 NBD structures, we noticed that the experimental TMD structures had a large range of conformations, with some resembling State 1 (average RMSD of 1.2Å to AF2) and others resembling State 3 (average RMSD of 3.2 to AF2). However, the experimental NBD structures were all quite similar to each other (average RMSD of 0.7Å to AF2; **Fig 5B**). This difference is likely because the TMDs perform most of the functional motion between conformations, moving like levers to draw the two NBDs together for ATP binding and subsequent hydrolysis. While the focus of this work is on domains and folds, we used whole Abcb1a structures to quantify how each fold type’s conformational heterogeneity combine to yield whole-protein RMSDs that are considerably higher than each domain (median of 5.9Å; **Fig 5C**). Therefore, investigating the structural similarities of proteins through their domains can partition RMSD differences to their root causes in domain dynamics, improving the mechanical understanding of protein functional motions.

### Folds exhibiting high conformational heterogeneity compared to high conformational Consistency are used differently across biological processes

Protein domains are typically modular components of larger molecular machines. Thus, each modular component has a classical function reused in different contexts. We quantified how protein folds distribute across biological processes and the intersection with our census of fold type flexibility. Surprisingly, we observed specific categories of gene ontology terms more frequently for proteins that contain fold types with high conformational consistency compared to proteins that contain fold types with high conformational heterogeneity. This finding clarifies that conformational regulation is more prevalent in certain biological processes than in others (**S5 Fig** in S1 Text). Specifically, we compared the 33 proteins that contain a domain with median RMSD > 2.5Å to AF2 references, to the 131 proteins that contain a domain from the lowest 10% of median RMSD. We found, for example, the proteins with heterogeneous domain are more frequently (15–33 more proteins) part of the enzymes that make post-translational modifications, regulate chromatin structure and remodeling, form multi-protein complexes, and participate in signal transduction processes. On the other hand, proteins with the lower decile domains are more frequently (25–100 more proteins) involved in immune system development and responses, and metabolism. Most count differences of at least six are statistically significant (p < 1×10^−3^ using the hypergeometric test). One specific instance is T-cell activation (p = 4.7×10^−7^). Thus, accounting for fold-specific flexibilities directly impacts research into these critical biological and cellular signaling processes.

## Discussion

We have leveraged large-scale structural databases to characterize experimentally-measured and fold-specific conformational heterogeneity and characterize how AI-based algorithms behave across this spectrum. This spectrum reveals biological insights because homogeneous folds versus heterogeneous folds are used by different types of proteins that drive immune and metabolic biology. Additionally, this spectrum informs about how to use the individual predictions returned by AI-based algorithms. Our study clarifies that gaining more significant biological inference from protein structures will require dynamic models or multiple models that span functional mechanisms.

To better understand the strengths and limitations of AI-predicted structures, we carried out domain-level comparisons with 176,257 experimentally determined protein domain structures. Comparison on such a large and heterogeneous dataset becomes necessary for a clear demonstration of the prediction characteristics of the tool. In addition, we minimized the potential disagreement between the predicted and experimental structures by considering the domains and not the whole proteins, which would otherwise have accounted for larger fluctuation because of the flexible inter-domain regions. AI-predicted structures are very accurate for most of the experimentally solved domains. Differences between experiments and predictions are partly because training data covers conditions that are amenable to crystallography, which is likely a subset of the biologic protein structural diversity. In the few cases where predictions ultimately failed to match experiments, one reason could be due to the lack of similar proteins in the training data. We found that the AI-predicted structures that most closely resemble experimental results have low surface area-to-volume ratios and high buried-to-exposed ratios, indicating a strong trend for predictions to be compact and globular. Current methods for predicting more extended and flexible conformations are more divergent from experimental results.

From their first uses, deep-learning architectures overestimated local interactions compared to predicting global architectures [42]. For example, the overrepresentation of secondary structure in the predicted model raises questions about how the coevolution of paired residues is used in contact map generation. Even though AF2 tried to minimize the overrepresentation of local interactions by using large MSAs for feature extraction and contact map generation, the secondary structure is still overestimated in our analysis. Thus, sequence-extrinsic features such as other molecules, metals, ions, ligands, or hydration water have major roles in fine-tuning secondary structure content. Also, we focused on differences in domain topology irrespective of sidechain position differences, which will also differ by environment. The CATH database hosts half a million domains, and we used 118826 that represent proteins with at least ten distinct experimental structures solved, so that we have a robust comparison of apparent protein dynamics. Considering these limitations is essential to successfully obtain a better estimate of the conformational heterogeneity of each protein domain and compared to their diverse experimental structural states. Accounting for molecular environment enables more flexible and extended conformations to be predicted that go beyond the one-sequence-one-model paradigm.

## Conclusions

In this work, we have calculated the spectrum of fold-dependent conformational heterogeneity and used AI-generated protein structure predictions as an objective and consistent benchmark. We found that predictions are close to experimental structures in most cases, yet divergent in others, identifying how best to interpret AI-generated structures in the context of protein form, flexibility, and function. We find that structure prediction algorithms have homed in on one specific conformation for each protein, rather than conformational averages, that closely matches experiments for most proteins and across protein folds. This accuracy simultaneously means they do not inform about the flexibility required for function. More significant biological inference will require dynamic models or multiple models that span functional mechanisms. This observation is consistent across protein folds, sequence lengths, liganded and non-liganded proteins, and more. Thus, it is a general feature at the intersection of current AI systems and protein fold knowledge, that must be considered when using data from these revolutionary algorithms.

## Supporting information

S1 Text The supporting text contains additional details and examples for our domain-level sub-clustering, structural and geometric calculations, and characterization of our dataset in terms of how termini and hinge motions affect calculations. (DOCX)

S1 Table We provide summary statistics for each fold type including the extent of conformational heterogeneity as calculated herein, multiple RMSD measures, the fraction of liganded structures, and the fraction of non-linear sequences used to make the fold. (XLSX)
