# Supplementary material for: Systematic analysis of the relationship between fold-dependent flexibility and artificial intelligence protein structure prediction
Source: PLoS One. 2024 Nov 26;19(11):e0313308. doi: 10.1371/journal.pone.0313308 (PMC11594405; doi:10.1371/journal.pone.0313308)

**Supplemental Text for:**

**Systematic Analysis of the Relationship between Fold-Dependent Flexibility of Artificial Intelligence Protein Structure Prediction**

**by Haque, et.al.**

**Domain clustering:** Domains defined by CATH mostly uniform and highly reliable. However, there are small fraction of domains that suggest the possibility of diverse domain belonging to the same segment of the protein (Figure 1A). To develop better understanding of the diversity of domains, we clustered all the domains based on the sequence followed by sequence length at the protein level, to cluster domains of similar nature as much as possible. Sequence based clustering was performed on all the 217402 domains using CD-Hit software [*Fu, L., Niu, B., Zhu, Z., Wu, S. and Li, W., 2012. CD-HIT: accelerated for clustering the next-generation sequencing data. Bioinformatics, 28(23), pp.3150-3152.]* with 95% identity cutoff. At this stage homologes and paralogues of 95% identical domains are clustered together. Furthermore, size-based clustering was performed to group similar sized domains from same segment of the proteins in one group (**Figure 1B & C**).

C

B

A


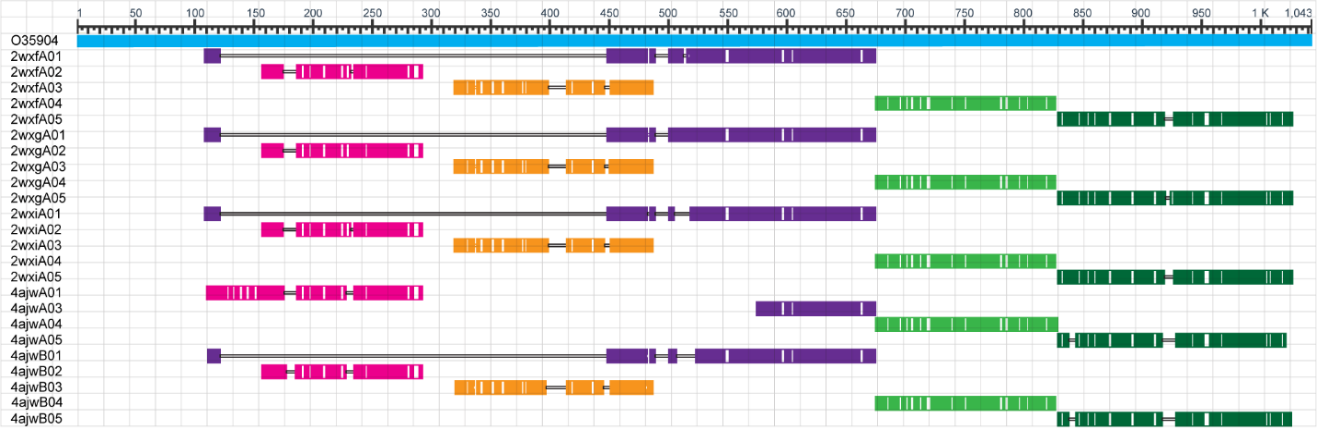

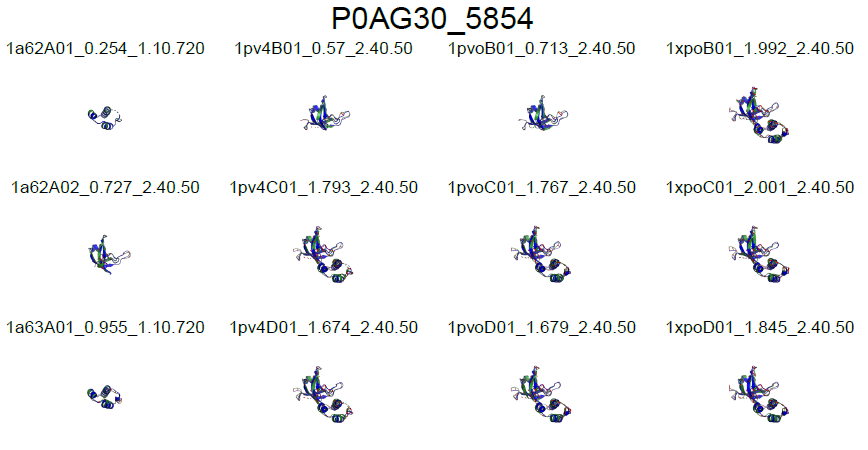

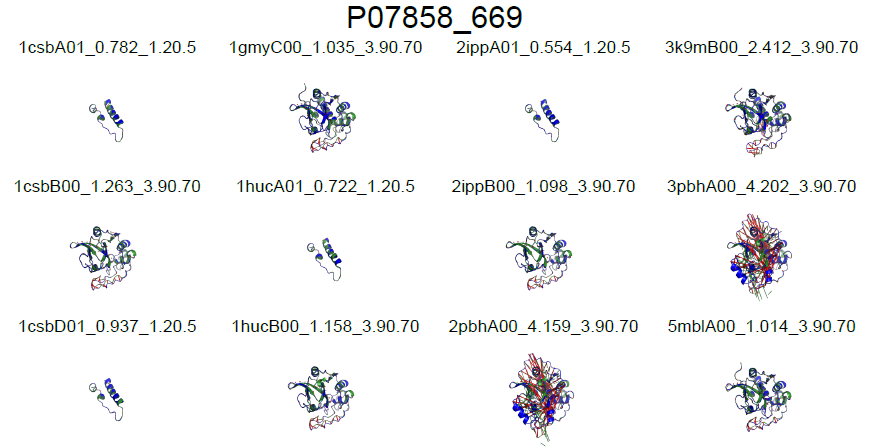


**Figure S1 A: Schematic representation of variation in CATH defined domain** (previous page)**:**  The example protein Phosphoinositide-3-kinase (Pik3cd) (Uniprot ID O35904, the first record): In our dataset show 127 domains are for this protein from experimental structures. For demonstration purposes we are showing only 24 domains. A full-length protein was classified into five different domains by CATH. If we ignore the small length overlap, we may recognize Dom1 (violet, ~5-51, 383-571), Dom2 (pink, ~52-187), Dom3 (orange, ~215-382), Dom4 (light green, ~572-723) and Dom5 (dark green, ~726-923). **B** and **C)** Shows pictorial representation of different domains from same segment of protein, such as, Transcription termination factor Rho of E coli and human Cathepsin B. respectively. The images are structure superimposition of the CATH domain (green color) and their AF2 structure (blue color) counterpart and the red colored line represent the structural deviation. The title of respective images consists of CATH ID, RMSD to AF2 counterpart, and the CATH fold ID.

**RMSD100 calculation:** The RMSD between two structures is dependent on their dimensions because of the nature of the equation $RMSD= \sqrt{\frac{\sum_{i} {d_{i}}^{2}}{n}}$ , where *d_i_* is the pair wise distance between the equivalent atoms in the aligned structures. The $RMSD100= \frac{RMSD}{1+ln \sqrt{\frac{N}{100}}}$ , where N is the domain length, is another metric to calculate the deviation between two structures which is independent of structure dimension [*Carugo O, Pongor S. A normalized root‐mean‐spuare distance for comparing protein three‐dimensional structures. Protein science. 2001 Jul;10(7):1470-3.*]. The normalization number 100 is used because it is supposed to be a favorable dimension of the protein fold [*Xu, D. and Nussinov, R. 1998. Favorable domain size in proteins. Fold. Des. 3 11–17*]. We computed the RMSD100 for all the CATH domains and their AF2 counterparts to understand the dependence of domain dimension in our observation.

**Structural metric computation:** The area and volume for AVratio is calculated using 3v: Voss Volume Voxelator [*Voss NR, Gerstein M, Steitz TA, Moore PB. The geometry of the ribosomal polypeptide exit tunnel. Journal of molecular biology. 2006 Jul 21;360(4):893-906.*] using default parameter for probe radius and grid. The secondary structure and residue-wise solvent accessible surface area (SASA)is computed using the tool STRIDE [*Heinig M, Frishman D. STRIDE: a web server for secondary structure assignment from known atomic coordinates of proteins. Nucleic acids research. 2004 Jul 1;32(suppl_2):W500-2.*]. The absolute residue-wise SASA was converted into relative SASA using the standard values of residues obtained from NACCESS software. [*Lee, Byungkook, and Frederic M. Richards. "The interpretation of protein structures: estimation of static accessibility." Journal of molecular biology 55.3 (1971): 379-IN4*]. BEratio is calculated by dividing the number of buried residues by the number of exposed residues, where a residue is considered buried if its 80% or above surface area is buried otherwise the residue is considered exposed. The secondary structure was divided into three classes such as, helix, sheet and all other secondary structure elements were considered loop. To evaluate gain and loss of secondary structure we computed the secondary structure for AF2 derived domain model also.

To further understand the role of metal ions and ligand atoms which were present during the development of experimental structures we obtained the experimental structure from RCSB and computed the number of residues involved in interaction with metal (distance cutoff for metal ion with any atom is 2.5Å), and with ligand (distance cutoff for ligand atom with any atom is 5Å and molecular weight cutoff for the ligand is 180 Da).

**Elimination of domains with extended termini:**

Multidomain proteins often rely on flanking residues to achieve different conformations or interactions, while the domains themselves are generally rigid with respect to extending flanking residues such as protein termini. Our data indicate that certain domains exhibit poorly defined termini, which are so extended that it is unreasonable to consider them part of the domain. To avoid bias, we excluded such domains from further analysis. Additionally, intradomain variations in experimental structures were observed in our data which we tend to analyze.

CATH definition of the domain of a protein is not unique as the same domain is represented by different boundaries. Furthermore, domains have extended termini which need to be marked and eliminated. So, we made an attempt to minimize such misrepresented structure by accounting for 3 scenarios: 1) With the impression that the domain is a compact structural unit of a protein we defined the terminal segment as non-domain segment if it doesn’t interact with the domain core. We quantify core interaction using atom-atom contacts of at least 4.5Å observed near the junction (i.e., residues 9-12 at the terminal). 2) To determine the non-domain segment, we evaluated only 10 residues (though it could be longer than 10 residue with the rational that if 10 residue at the terminals are identified the longer one will automatically be identified) at the terminals and computed the Euclidean distance between all the atoms of the domain. 3) If the 10 residues in N-terminal were unable to make four contacts of at least 4.5Å then then we mark them as extended termini and remove them from further analysis.

**FATCAT, Simple and complex hinge motion:**

We applied the FATCAT hinge correction algorithm (installed on our local high-performance computing cluster) [*Ye, Y. and Godzik, A., 2004. FATCAT: a web server for flexible structure comparison and structure similarity searching. Nucleic acids research, 32(suppl_2), pp.W582-W585.*] to categorize the different conformations as those that can be easily accounted to simple hinge motions, complex hinge motions, and more nuanced rearrangements that cannot be considered as hinge motion at all. We applied FATCAT to the subset of our dataset with extended termini and perfect alignment of CATH domains with their AF2 counterparts. FATCAT identifies one or multiple pivotal residues around which, if one part is fixed and the other is rotated, proper alignment between two structures can be achieved. This correction is performed through transformations involving one or multiple twists. For simplicity, we classify domains as exhibiting simple hinge motion if FATCAT transforms the structure with one or no twist, and as having complex hinge motion if the transformation requires two or more twists.

However, we observed that even for simple hinge motions, FATCAT's RMSD calculations ignored unaligned residues and reported only minimal RMSD of the most optimally superimposed sub-structure. Additionally, even with corrections involving a higher number of twists, the RMSD remained minimal (**Figures S2 and S3**). Figure S4 further shows that only a very small fraction of domains are marked as having complex hinge motion with higher RMSD, while most domains with simple hinge motion are transformed to have low RMSD values. This suggests that FATCAT's RMSD calculations are predominantly based on residues that align properly, resulting in small RMSD values.

Therefore, although FATCAT effectively recognizes both simple and complex hinge motions, it is not suitable for this study, which aims to measure structural differences in domains arising from intradomain simple hinge motion.

**Figure S4:** A) Distribution of ratio of fraction of buried to exposed residues and B) ratio of surface area to volume of the domain. The data is plotted with RMSD additionally the domains are marked with fraction of simple hinge motion (solid color shows zero or one twists), and complex hinge motion (the fraction of domains with two or higher number of twists shown by shaded segments).


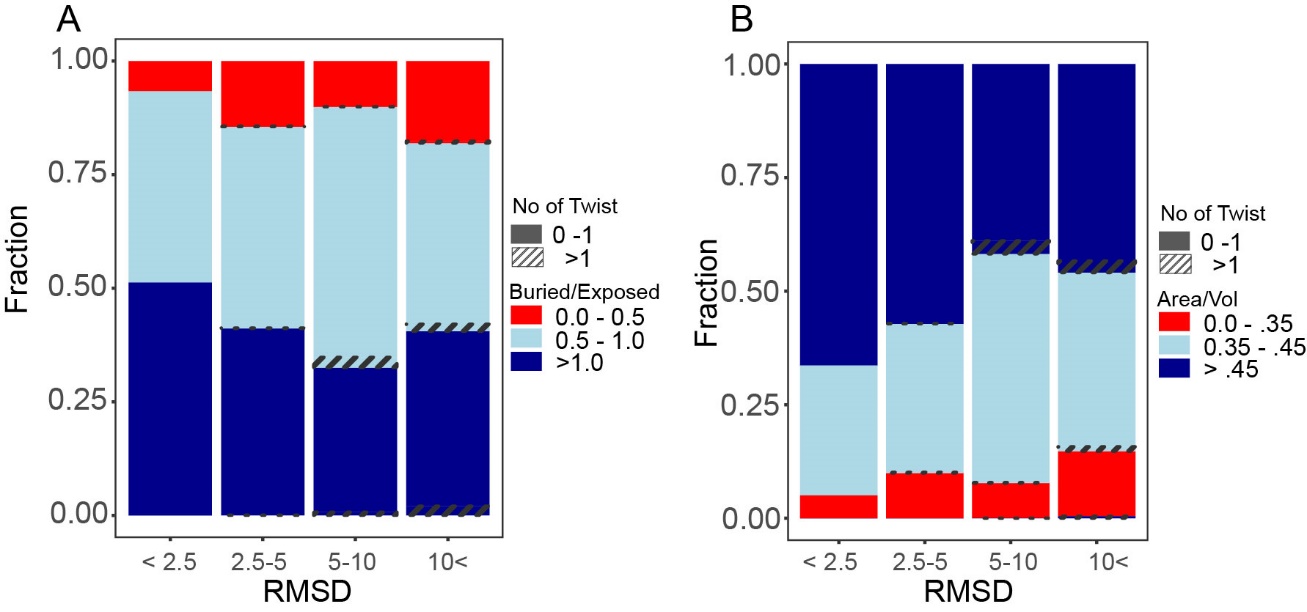


**Figure S3:** Distribution of RMSD and FCrmsd of domains and their predicted counterparts. The inset shows that FCrmsd tops at 5Å whereas RMSD has values even higher than 10 Å.


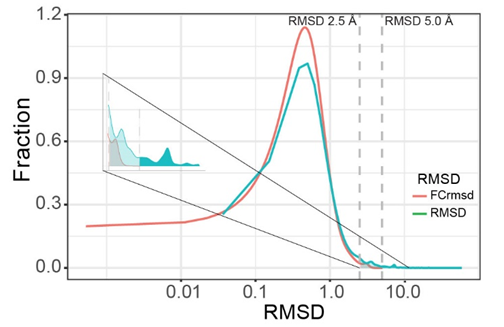


**Overrepresentation of GO terms between proteins that contain the most homogeneous versus heterogeneous folds:**

Overrepresentation of GO terms are calculated by subtracting number of go terms associated with unique protein in the 10% of fold group at lower end of median RMSD and fraction of fold group above median RMSD of 2.5Å (**Figure S5 A-C**). Similarly, GO terms difference for unique proteins were also subtracted from median FATCAT RMSD (FCrmsd) ordered data (**Figure S5 D-E**).

It is interesting to note that Overrepresented molecular function such as protein homodimerization activity, ATP-dependent protein folding chaperone, unfolded protein binding, cadherin binding, disordered domain specific binding, heat shock protein binding, histone deacetylase binding, magnesium ion binding, protein kinase binding, ubiquitin protein ligase binding, ATP binding are among proteins that are flexible in nature.

**Figure S5: Biological significance of RMSD distribution of domains in folds.** Proteins represented by domains in RMSD distribution of folds, shown in **Figure 3A**, are also shown for their presence in different GO categories (see next pages): (**A & D**) Biological process, (**B &E**) Cellular components, and (**C & F**) Molecular function of data in median RMSD (**A-C**) and FATCAT median RMSD (**D-F**) order. The overrepresentation of certain proteins in a GO category is calculated by subtracting the number of proteins in the lowest median RMSD decile (initial 10 % of folds or median RMSD lower than 2.5Å) from the highest median RMSD decile (top 10 % of the folds) in a given category. The GO categories possessing fold representative proteins in the lower and upper deciles are shown in blue and red colors, respectively.

Figure S5 A


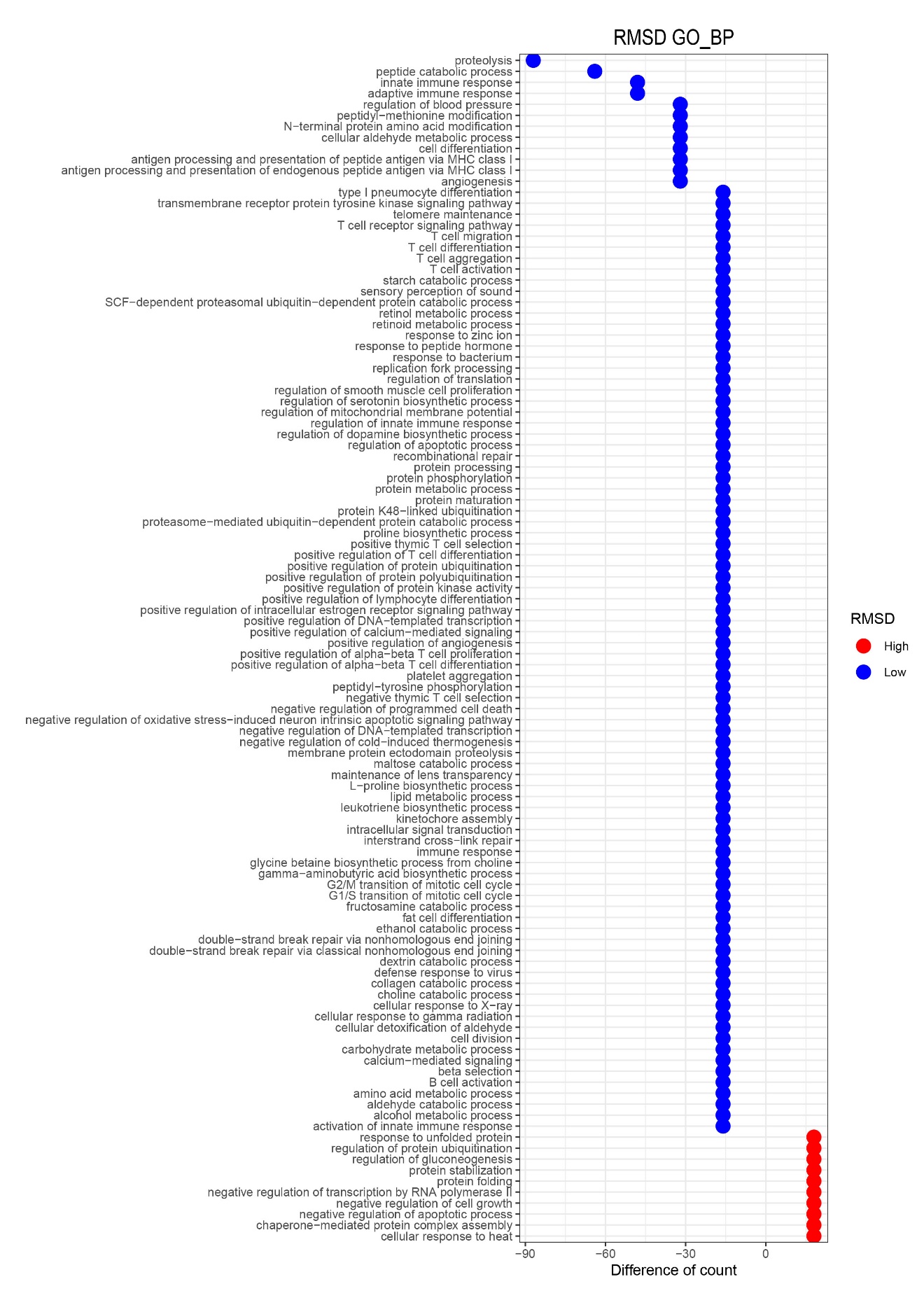


Figure S5 B


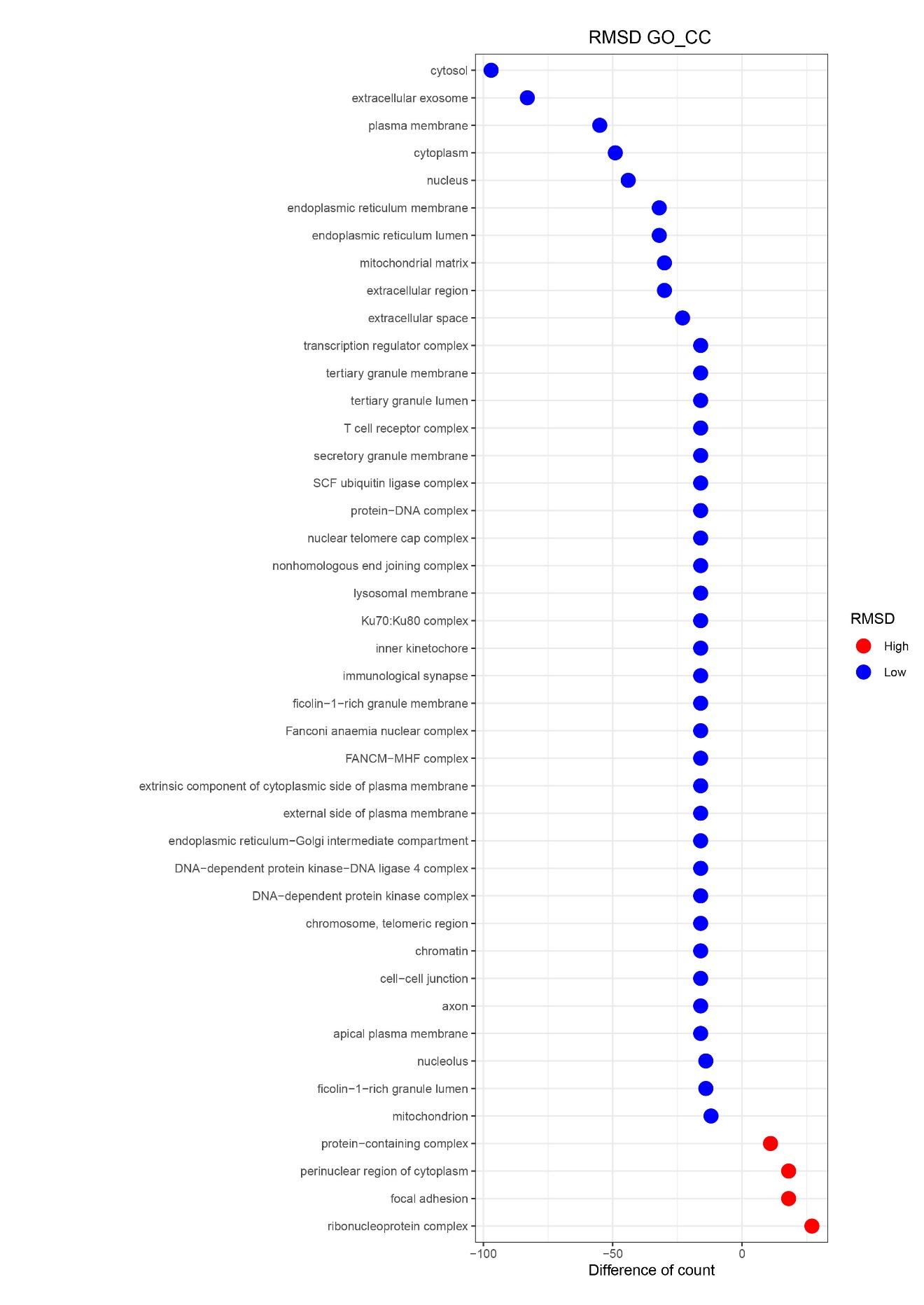


Figure S5 C


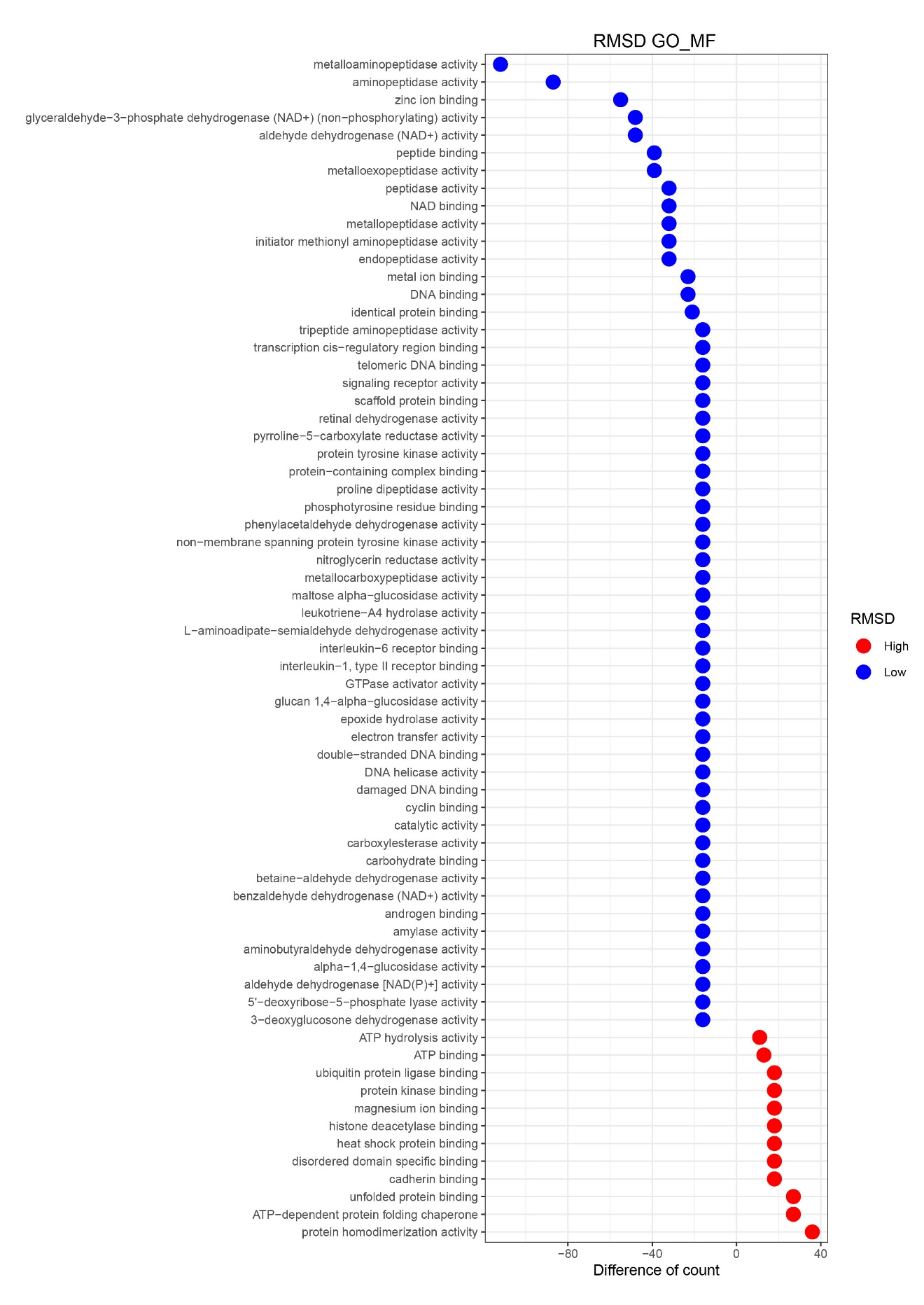


Figure S5 D


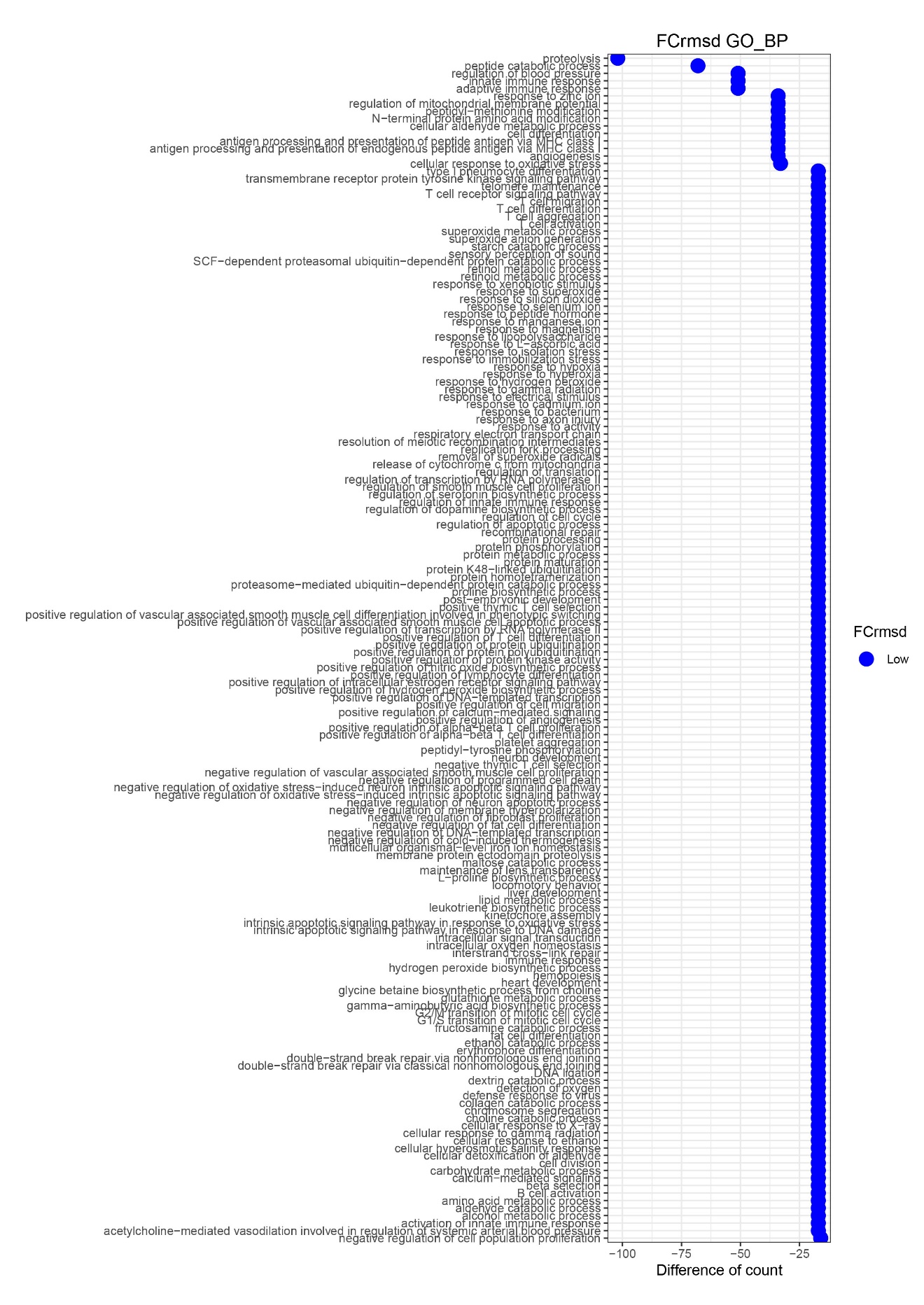


Figure S5 E


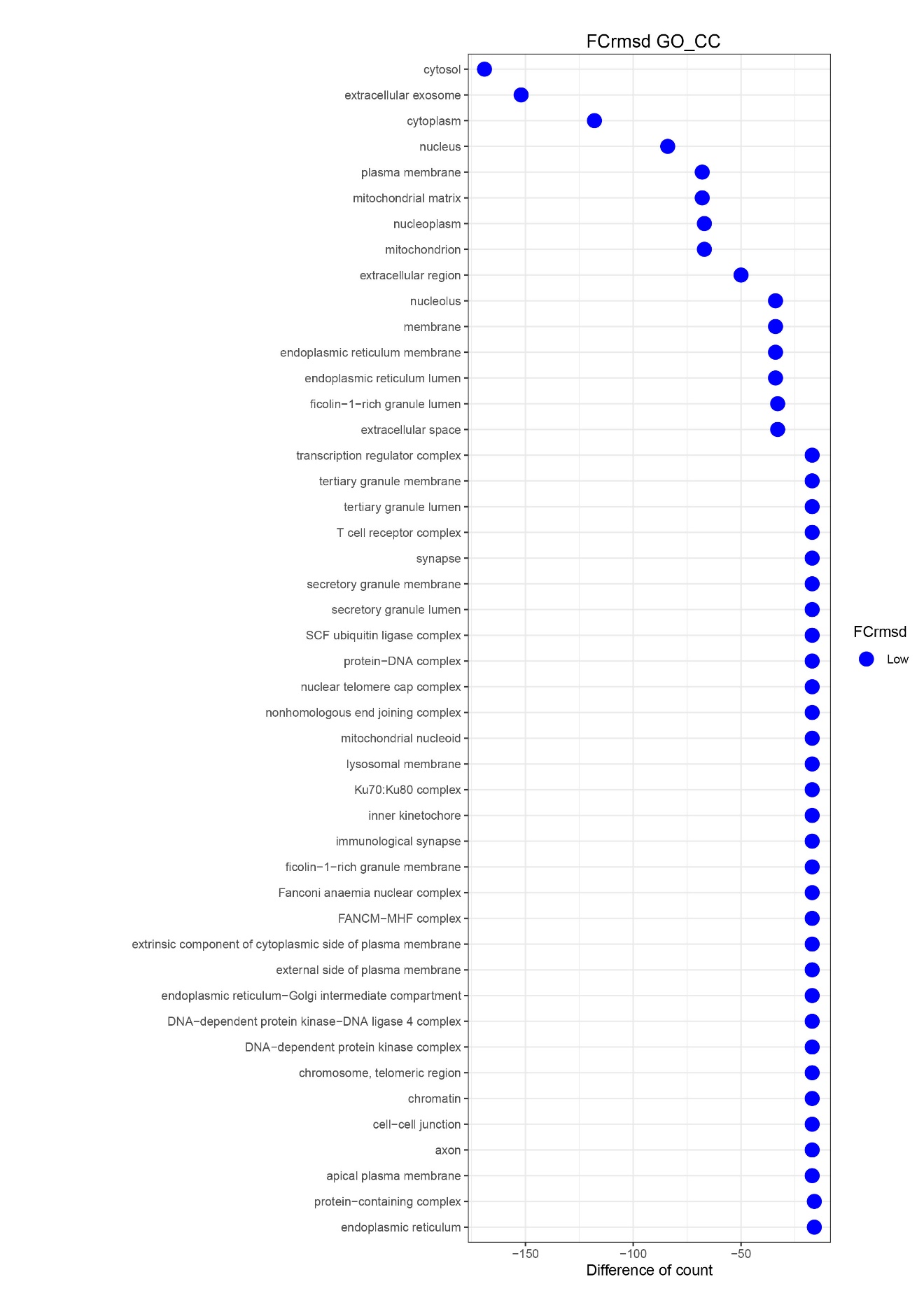


Figure S5 F


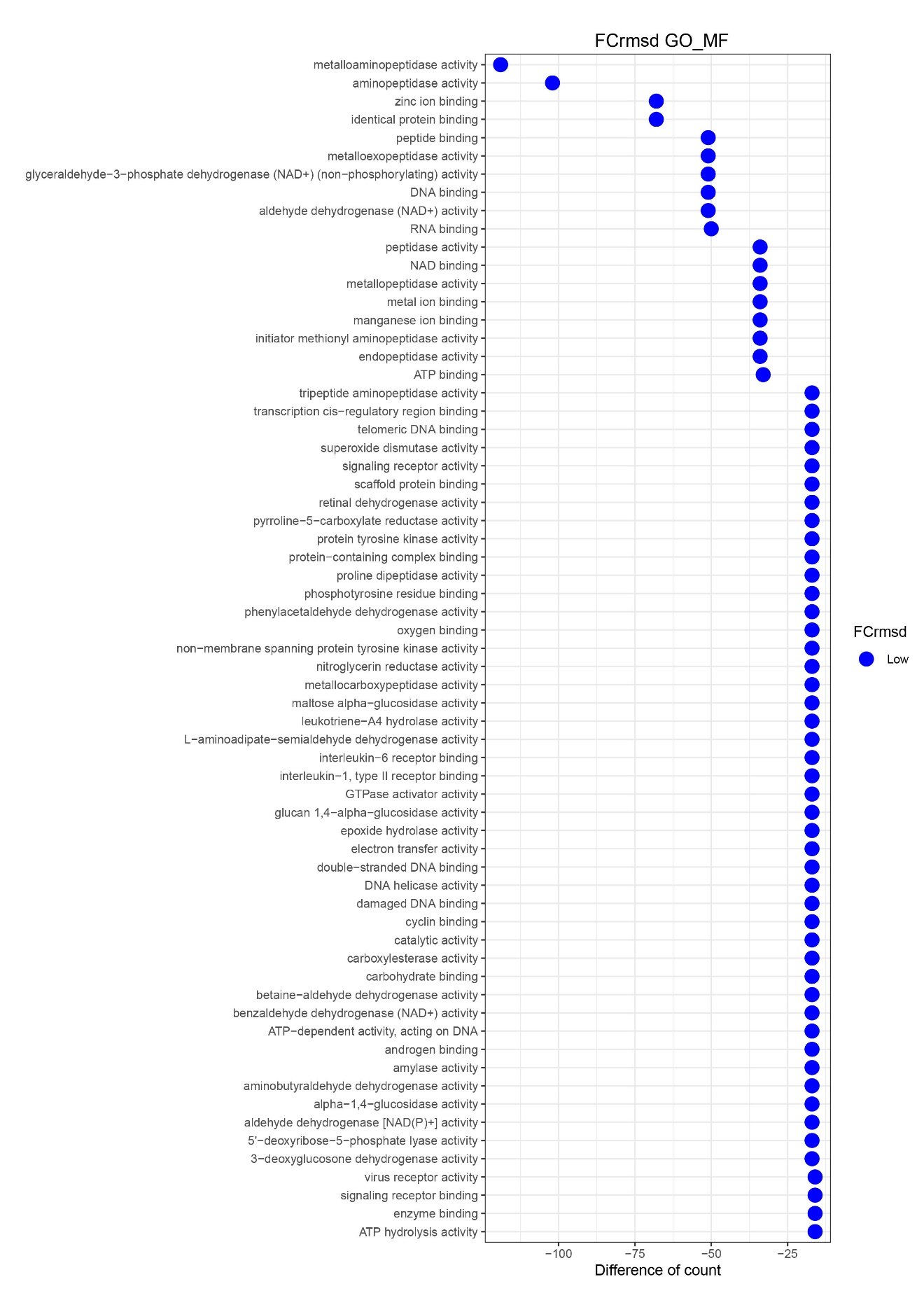

Supplement: S1 Text — (DOCX) [file pone.0313308.s001.docx]
